# Methodological Challenges in the Economic Evaluation of Occupational Health and Safety Programmes

**DOI:** 10.3390/ijerph15112606

**Published:** 2018-11-21

**Authors:** Jonas Steel, Lode Godderis, Jeroen Luyten

**Affiliations:** 1 Leuven Institute for Healthcare Policy, KU Leuven, 3000 Leuven, Belgium; jeroen.luyten@kuleuven.be or ligb@kuleuven.be; 2 Environment and Health, KU Leuven, 3000 Leuven, Belgium; lode.godderis@kuleuven.be; 3 IDEWE, External Service for Prevention and Protection at Work, 3001 Leuven, Belgium; 4 Department of Health Policy, London School of Economics & Political Science, London WC2A 2AE, UK

**Keywords:** occupational health and safety, economic evaluation, methodology, workplace, productivity

## Abstract

An emerging issue in occupational health and safety (OHS) is that interventions increasingly have to demonstrate that they offer sufficient value for money. To this end, the last decennia have seen more and more economic evaluation methods being employed in this field. However, several recent publications have indicated that many of the published studies suffer from important shortcomings. This paper aims to highlight difficulties in assessing the value of OHS by use of current economic evaluation methods. First, a summary framework presents an overview of the costs and benefits relevant for OHS interventions. Next, three elements from this framework are selected that are at the same time crucial to OHS value, but also challenging to measure and monetise: Effects on worker productivity, ‘intangible’ benefits, such as reputation effects, and the influence of the broader legal–fiscal context in which an intervention takes place. The following sections then discuss the following research questions for each of these elements: Why is it difficult to exclude these factors from OHS economic evaluations? Why do they pose a challenge to the quality of economic evaluations in OHS? How can they be included, and what are the known advantages and disadvantages of the methods to measure these factors? Future work should investigate (and standardise) better methods to include these elements.

## 1. Introduction

Matching the continuous emergence of new treatments and technological changes with limited resources is a crucial challenge in the healthcare domain [1]. This delicate exercise makes it necessary for decision-makers to assess opportunities critically, in order to ensure that limited resources are invested in interventions with effects that merit their costs, without foregoing better alternatives. Economic evaluation can help to make these difficult decisions by comparing the costs and effects of different interventions. The last decades, publications related to economic evaluation have been strongly increasing in number.

An analogous increase of economic evaluation is noticeable for occupational health and safety (OHS) programmes—interventions that focus on improving the health, well-being, and safety of employees at the workplace (e.g., hazard control, return to work, screening, or health promotion at work). This is logical, as—although important improvements have been made—OHS faces similar challenges: Limited resources need to be reconciled with a considerable burden of OHS injuries and diseases [2], an aging working population [3], and upcoming health hazards, such as nanomaterials or e-waste [4]. Resources are perhaps even more pressurised in OHS, given the competitive enterprise environment in which OHS is often situated. The need for economic evaluations in OHS is therefore substantial [5].

However, several publications have highlighted that many of these economic evaluation studies are lacking sufficient scientific quality [6]. To some extent, this is because the measurement and attribution of the effectiveness of OHS interventions is inherently more challenging than it is in, e.g., the domain of pharmaceutical evaluation. For instance, it is more difficult to implement randomised controlled trials (e.g., because of legal requirements) [7,8], a wide range of health and non-health outcomes need to be measured [9,10], the stability of measurement units over time can be problematic (e.g., because firms can have “high rates of closing, merging, moving, downsizing or restructuring” [11]), there is often a long latency period before (positive or negative) effects can be observed, and OHS possesses multiple features of complex interventions and systems [12,13]. In addition, some researchers have observed that many studies diverge from the ‘reference case’ (e.g., as described by Drummond [14]) in how economic evaluation should be executed and reported: Underreporting of the employed methodology and context, not including all relevant costs and consequences, incorrectly valuing them, insufficiently accounting for uncertainty or justifying assumptions [15,16,17,18].

In this article, we want to extend upon these areas for improvement by arguing that the current methods of economic evaluation are at times ill-devised to evaluate OHS programmes. More precisely, calculating the economic benefits of OHS interventions typically requires including several factors that are notoriously hard to measure and monetise. Most of these methodological shortcomings are well known, but in the context of OHS they become essential, as they concern factors that are (in contrast to other domains) of key value in OHS programmes.

## 2. Methods

As a first step, the authors present an overview of the costs and benefits associated with OHS investment. This is based upon frameworks of economic evaluation [14], of economic evaluations in OHS [3,19,20], of case studies identified by previous reviews [21,22], and upon descriptions of the decision-making process in implementing OHS [23]. The result of this exercise is presented in Section 3. The second step selects factors from the framework that contribute to a lack of quality in OHS economic evaluations. We focused upon factors that are an important source of value in an OHS setting (but are typically overlooked or less important in other healthcare domains) and are challenging to measure or monetise. Other challenges that are not OHS-specific (e.g., how to incorporate health effects, the use of discounting for costs and effects, how to measure or value grief and suffering of relatives), or OHS specific factors that are less challenging (e.g., avoided damage to factory equipment) are beyond the scope of this article.

Three such factors were identified: The effects of OHS on health-related productivity, intangible benefits of OHS, and accounting for the fiscal–legal context. Section 3.1, Section 3.2 and Section 3.3 investigate these factors in turn by answering three research questions: Why is it difficult to exclude these factors from OHS economic evaluations, in contrast to economic evaluation in other domains? Why do these factors pose a challenge to the quality of economic evaluations in OHS? How can these factors be included, and what are the known advantages and disadvantages of the methods to measure these factors?

## 3. Three Challenges of Economic Evaluation in Occupational Health and Safety

**Figure 1:**
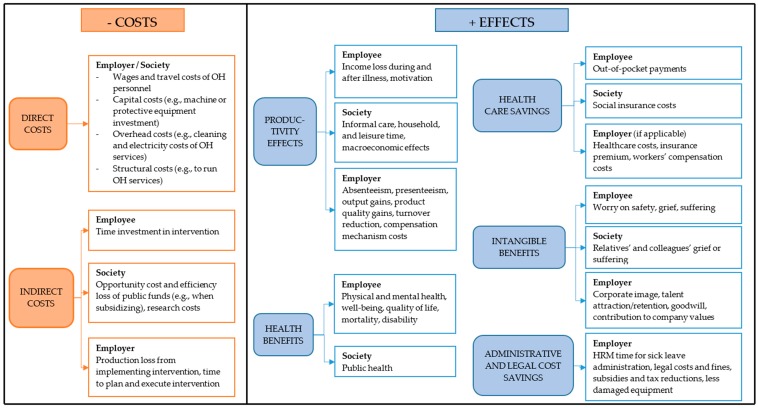
Costs and effects of occupational health and safety interventions, by cost and effect category and by perspective. [Blue = intervention benefits or effects; Orange = intervention costs, OH = occupational health, HRM = Human Resource Management].

Figure 1 provides a graphical overview of OHS costs and benefits. It does not aim to provide a comprehensive or exhaustive overview, nor to describe what should (or should not) be included, but rather serves to highlight the differences of OHS with other economic evaluation domains. The left panel illustrates the direct and indirect costs of OHS interventions from the perspective of employees, employers, and society. The right panel uses those same perspectives, and categorises effects of OHS programmes into productivity benefits, health benefits, health care savings, intangible benefits, and administrative and legal cost savings. Note that the societal perspective takes into account the relevant costs and benefits for all stakeholders (employees, employers, taxpayers, etc.), but without double counting. For instance, productivity effects for the employee (income loss) and the employer (output loss) are closely related and should not be included twice. Since many OHS programmes rely on group-based decisions (e.g., by representatives of unions, employees, employers, occupational health specialists), it is often important to include multiple perspectives in the analysis.

What comes to the fore in comparison to other healthcare domains is the addition of the employer perspective and the particular costs and effects that accompany it. In evaluations in other settings, a patient or healthcare payer perspective is customarily used, which puts the largest weight in the decision-making process upon health effects and healthcare costs. In contrast, the employer perspective is more commonly adopted in OHS [21,24]. This is logical, as managers play a pivotal role in the funding and implementation of OHS interventions [25]. In turn, this reduces the weight of direct health effects (compared to other settings), and makes other (groups of) factors that are typically overlooked in other healthcare domains more important. The sections below discuss the challenging nature of three such factors: Worker productivity, intangible benefits (e.g., firm reputation and safety), and effects linked to the legal–fiscal context (e.g., employee indemnity claims, workers’ compensation expenses [26], or financing OHS through subsidies or tax reductions).

### 3.1. Effects of OHS on Worker Productivity

Productivity loss is often equated with the fact that healthier individuals will show less absenteeism and presenteeism (reduced performance at work). However, it might be more precise to see it as lower efficiency in production, which means less output is obtained from a given set of inputs [27]. In economic evaluations in other healthcare domains, the inclusion of productivity losses (sometimes referred to as an indirect cost [28]) remains controversial, with some guidelines advocating their use (e.g., Sweden and the Netherlands), while others advise to exclude it (e.g., the UK, Belgium, and New Zealand) [29]. Amongst other reasons, it is put forward that productivity losses are not a natural element of healthcare evaluations, and that their in- or exclusion only serves to boost cost-effectiveness ratios if so desired [30]. In contrast, the central role of the employer as decision-maker makes it difficult to follow this argument in OHS. Notwithstanding their controversy in other healthcare domains, productivity losses are one of the core reasons why employers choose to implement OHS interventions beyond required legal standards. A recent review indicated several evaluations even take productivity up as the sole benefit in the analysis [21]. As such, it would be hard to defend the systematic exclusion of productivity in OHS, and it makes the precision of its estimation of great importance.

However, the measurement and monetisation of productivity is complex [21]. First, at least seven different valuation methods are available (output-based methods, human capital approach, friction cost approach, multiplier approach, productivity in natural units, US panel approach, and making use of workers’ compensation expenses), and there is as of yet no consensus on which one should be used in which circumstances [21]. Second, it is not straightforward to obtain qualitative absenteeism and presenteeism data. Objective sickness days data are often incomplete (e.g., human resource data are not always accurate, insurance data often focus upon compensated absences), while subjective (self-reported) absenteeism (and presenteeism) is limited due to recall bias. Furthermore, the magnitude of time-loss estimates depends on the chosen survey, and not all surveys have been validated [31,32]. Third, to monetise absenteeism and presenteeism, an adequate price weight (e.g., the employee’s wage) is needed. Depending on the guideline followed, the composition (are secondary benefits included?) and type (job, individual, or industry wage?) of the price weight differs, and requires additional data. Finally, if one wants to ensure that this monetary estimate reflects real loss in productivity, adjustments are needed. For instance, a (national) friction period must be estimated (only available in the Netherlands and the UK [33,34]), or job-dependent multipliers should be used to indicate the absence of perfect substitutes, the effects of team production, and the penalty of not reaching an output target [35,36].

Since there is as of yet no consensus on how to measure productivity, we recommend on the one hand to follow (national) guidelines (e.g., Oostenbrink et al. [37] for the Netherlands), as this contributes to standardisation. On the other hand, it has been observed that the appropriateness of (valuation) methods may depend on the intervention, outcome, perspective, time horizon, and study design of the evaluation [21,38]. We therefore suggest to include productivity separately in economic evaluations, and to perform analyses using several methods. Given the strong differences between the two [38], one could argue that, at minimum, the human capital and friction cost approach should be applied, but sensitivity analyses with the multiplier approach and including costs of compensation mechanisms can be added. Since clear instructions on how to perform the different methods are available (e.g., the Dutch Costing Manual [39] for the friction cost approach), this should be a feasible opportunity. For instance, Van Wier et al. [40] use both the human capital and the friction cost approach from a societal and employer perspective. Regarding productivity measurement, using objective data on sick leave that are separable from other absences (e.g., holidays) is often the most precise. If such data are absent, several validated and reliable instruments are available (e.g., PRODISQ, HLQ, WHO-HPQ, iPCQ) for self-reported absenteeism [21,41], although the recommended recall periods of these instruments should be respected. Presenteeism is more controversial, and is best measured by self-report (unless production data are available, e.g., number of files handled per hour in a phone centre), e.g., using the iPCQ, or HLQ [21,42]. Since estimates of presenteeism can differ across instruments, it seems best to follow the recommendations of guidelines (e.g., the Netherlands advises the use of iPCQ).

### 3.2. Intangible Benefits of OHS

In economic evaluations, the term “intangible costs” (or “intangible benefits”) is used to indicate costs and consequences that are difficult to measure and monetise [14], although there is not always a clear consensus on what this encompasses. Some examples in a clinical setting are effects on the social context (e.g., aggressiveness caused by substance abuse [43]), pain and grief of family and friends, or effects upon quality of life (e.g., pain, joy, physical limitations) [28,43]. Because of the methodological difficulties posed with quantifying or valuing these effects, most studies do not include them. As Figure 1 indicates, OHS programmes lead to several intangible costs and benefits and these are a key part of the value offered by OHS programmes. Some of these occur in other healthcare domains—quality of life effects, pain and suffering—but OHS also has some unique intangible consequences.

First, investments in OHS can affect a firm’s labour pool. A good reputation on the labour market can contribute to attracting more and more talented workers, and can help to retain key employees. Accumulating the right combination of human capital could in turn help firms to obtain a competitive advantage [44,45]. Given the current workforce demographics (e.g., retirement of baby boomers), and especially in sectors or occupations with high turnover rates (e.g., teachers [46] or nurses [47]), this becomes an important factor to consider.

Second, investments in OHS can have an effect upon costs associated with a bad reputation. ‘Sweat shops’ [48], unequal treatment of migrant workers [48], textile factory accidents [49], or excessive overwork [50] can catch public attention and affect a firm’s image, and thus possibly hit customer brand loyalty or sales. A survey among large UK companies (the yearly Captains of Industry Survey) also indicated this as a concern among managers: 79% of the senior directors saw health and safety as having a tangible impact upon corporate reputation [51], while later versions of the survey (e.g., in 2012) noted reputation or company brand as a key factor to stand out as a company [52].

Third, many investments in OHS will affect the risks and safety of the workplace. Given its different definitions [53] and disciplinary contexts [54], the challenging nature of risk manifests itself on several levels in occupational health and safety. From the viewpoint of risk analysis, challenges rise in the appropriate assessment and management of hazards in the workplace to minimise risks on employees’ health and safety. In essence, the focus of these exercises lies upon mapping out the likelihood of hazardous events and the severity of the health burden they cause. However, questions are rising on whether such probability-based approaches are too narrow for risk assessments. Instead, some maintain the view that probabilities cannot reflect all the knowledge and information on uncertainty that is needed [53,54]. For instance, Paté-Cornell [55] is of the opinion that “perfect storms”—rare conjunctions of factors with known probabilities—require systemic methods that pay attention to dependencies among factors, and “black swans”—rare and difficult to anticipate events—call for proper attention to (less tangible) signals and precursors. In addition, Aven [54] suggests that qualitative methods are needed to ensure all aspects (e.g., social and political aspects, value judgements such as the (pre)cautionary principle) are taken up in decision-making. In this sense, both can be seen in the wider trend towards risk-informed decision making—seeing risk analysis as a decision support that aims to inform decision-makers—rather than risk-based decision making—(quantitative) risk analysis as the sole basis for decision making [56].

From the viewpoint of decision modelling [57], a challenge of safety in OHS lies in making adequate decisions (e.g., to implement a programme or not) given a scarcity of high-quality evidence on OHS interventions’ cost-effectiveness. This scarcity multiplies the risk of making a wrong decision when choosing whether to implement an intervention, and thus the associated costs of making the wrong decisions (the opportunity cost of foregone funds). In situations of high stake investments with high uncertainty, it can therefore be useful to not only compare the costs and benefits of implementing or not implementing a programme, but to also compare it with postponing the decision until more evidence is available (a value of information approach [57]).

Finally, in the field of economic evaluation, there is the challenge of how risk and safety should be taken up in the analysis. A common counterargument against including effects on safety is that it can lead to double counting, as increases in safety overlap with the health improvements that come along with them (e.g., fewer accidents or fractures). However, in OHS, safety seems to have benefits beyond their eventual translation into health improvement. A first observation is that employees worry when there is a lack of safety. Conversely, knowing that everything has been done to ensure a safe work environment might reduce these concerns, giving a clear additional benefit for employees. Next, insights from behavioural economic and psychological literature indicate individuals have other attitudes towards risk and safety than towards health. For example, they value high-impact incidents that occur infrequently (e.g., a terror attack or large accident) as disproportionally more burdensome than small incidents that occur frequently (e.g., getting influenza) [58], even though the aggregate impact of the latter can be bigger (in terms of both total health impact and associated costs). If economic evaluations do not take these attitudes into account and look only at the expected health effects of an OHS intervention, return-on-investment estimates might not result in the right prioritisation of programmes (i.e., in line with employees’ preferences).

In contrast to clinical settings, a study by Miller and colleagues [59] indicates several of these intangible benefits are influential in managers’ investment decisions in OHS: Interviews point out their choices rely on a combination of financial, ethical, social, and legal factors, as well as intuitive arguments on people management or corporate reputation. An economic evaluation aiming to aid employers in their decisions could therefore incorporate these effects where they are deemed important. At the moment, a first step to include these effects (upon the labour pool, firm reputation and sales, less tangible signs of risks, employee worry, and attitudes towards risk) is to highlight their existence to decision-makers when they are likely to change because of the intervention. However, this leaves the weighting of these factors in the decision entirely in their hands. Second, as indicated by Drummond et al. [60], “intangible” can be a misleading term, as these effects can still be measured and included in analyses. For instance, quality of life effects can be assessed through surveys (e.g., through the SF-36 health survey [61]), and other intangible costs are—in principle—measurable through willingness-to-pay estimates, indicating how much money people are willing to sacrifice for avoiding these intangible burdens. A more challenging possibility is thus to estimate intangible effects directly (e.g., surveys measuring employees’ or managers’ perceptions of labour pool effects or willingness-to-pay for risk reductions) or indirectly (e.g., through their effect upon sales or productivity). Finally, instruments exist that try to take intangible OHS effects into account, such as sustainability reports (a company reporting publicly on its economic, environmental, and/or social impacts) [62] (although these have received criticism [63]), instruments that measure employee motivation [64], job satisfaction [65], firm reputation [66], or perceived safety climate [67]. While these instruments often originate from the OHS community, they have not been regularly considered as outcomes in economic evaluations of OHS. In general, more methodological work is needed to ensure these issues can be taken up consistently.

### 3.3. Accounting for the Legal and Fiscal Context

OHS is, more than other healthcare domains, set in a complex social, legal, and fiscal context that strongly diverges between countries. Dekker et al. have pointed out the far-going bureaucratisation of safety in many countries, including “*increases in rules, paperwork, costs, time drain, safety people involved, and compliance expectations that are insensitive to the demands of front-line activities*” [68]. While acknowledging the safety gains, rationalisation, order, and efficiency that safety administration has brought, they claim the yield of further bureaucratisation is declining or plateauing in many industries [68]. Regardless of their effectiveness, extensive safety regulations complicate economic evaluations of occupational health and safety programs as they entail a significant administrative burden and time investment for the employer, who may therefore be less inclined to devote time to other programs. In addition, safety regulations differ across industries and countries, posing challenges to the generalisability of research results.

On the cost side, OHS programmes are often subsidised by the government, or tax reductions can be applicable to purchases related to OHS. A report by the European Agency for Safety and Health at Work (EU-OSHA) indicates most European countries take measures to incentivise OHS investment [69]. While tax incentives were rather rare, most EU countries make use of insurance-related incentives (e.g., a bonus–malus system for insurance premiums based on a companies’ accident rates), and nearly every EU country used funding schemes for OHS (e.g., subsidies or grants for purchasing materials and tools, or to implement OHS management systems) [69]. For instance, in the German butchery sector, a combination of positive premium variations (when investing in occupational safety) and funding schemes for safety and health, was introduced [69].

On the effects side, an important part of the benefits of OHS is avoiding costly occupational injury, disease, or death. Without countermeasures, the burden of not investing in OHS falls largely on the employee and their families, as they would bear the health and income consequences (e.g., loss of wage or rise in healthcare costs). For this reason, several systems have been devised that help to relieve this burden upon employees, by shifting (part of the) costs back to the employer or to society. These systems range from worker compensation systems (e.g., USA or Canada), sick pay, taking OHS compensation up in the general social insurance system, accident compensation, (employer organised) disability insurance, to employers’ liability (and the possibility to legally claim compensation) [26]. The exact implementation of these systems also differs. For instance, while the Netherlands requires up to two years of sick pay to be paid by the employer, this is only up to 30 days in Belgium, with social insurance taking up a much larger portion of the payments. Each system, thus, has very different consequences on who (employee, employer, society) bears what portion of the burden of occupational disease and injury. Some systems will result in little reimbursements to employees (e.g., due to underdeveloped OHS legislation), others will emphasise the responsibility of the employer and ask them to compensate the majority of the costs to the employee, while again other systems will rely more strongly upon society’s solidarity by shifting OHS costs to the general social security system.

In turn, these differences can strongly affect the economic benefits from investing in OHS interventions, even when exactly the same intervention is carried out [22]. An economic evaluation of an OHS programme, aiming to indicate the expected return to a stakeholder relative to its required costs, should therefore carefully assess and report what OHS compensation systems are in place to correctly determine the consequences of OHS investments and disentangle the burden and benefits of each stakeholder. The relevance of this context is obvious from an employer’s perspective as, in this case, fiscal–legal effects are the most tangible. However, ideally, economic evaluation involves taking a societal perspective, as this ensures that the effects upon the healthcare system as a whole (and thus taxpayers) are taken into account. This can be complicated, as particular money streams (e.g., subsidies or fines) must be seen as transfers rather than costs. Precisely performing and reporting this exercise becomes even more important when one realises economic evaluations in one setting are often used to inform decisions in other settings, since many choices (rightfully) rely on prior research rather than re-evaluating the same programmes.

## 4. Discussion

Economic evaluation can be a valuable tool for decision-makers in OHS facing resource allocation tasks, as it provides detailed information on the costs and outcomes of interventions. When carefully executed, it provides useful estimates of the return-on-investment of competing programmes. However, several publications have outlined that published studies are often insufficiently reliable and that better economic evaluation studies are needed. This article aimed to point out that this is a difficult task, as the available methods of economic evaluation are often ill equipped for the particular OHS context. It did so by exploring the challenges in correctly measuring three effects that are typically not encountered (or deemed less relevant) in other healthcare domains: Productivity losses, intangible effects, and effects of the legal and fiscal context. As such, it contributes to previous work [8,15] that offered broader methodological guidelines in OHS, and it answers to the call for more research on economic evaluation in occupational health [70].

The three elements discussed above remain difficult to address. They are less relevant in other contexts, which makes them less of a methodological priority. However, OHS does not always fit into the standardised approaches for other disciplines, as these elements cannot be as readily excluded because of their importance in the decision-making process (our first question).

Instead, they remain challenging factors that need to be included (our second question): Estimating productivity loss knows many practical difficulties (how to adequately measure absenteeism and presenteeism) and methodological disagreements (what should be included and how should it be monetised). Intangible benefits are by definition difficult to grasp and monetise within an economic evaluation. Nevertheless, they indirectly lead to tangible consequences: Effects upon the labour pool and firm reputation can lead to changes in firm profit, picking up less tangible signs of risks can avoid costly accidents, and reducing an employee’s worries could benefit work satisfaction and production. Finally, the diversity of the legal and fiscal context and the bureaucratic burden that often accompanies it complicates research and the generalisability of its results. Each of these elements, thus, poses a barrier for economic evaluations of OHS to attain a higher quality.

While we referred to recent methodological work that tries to resolve these matters, there is as of yet no clearly dominating methodology (our third question), and all of these elements would benefit from more fundamental research. Productivity needs methodological work on estimating friction periods for other countries, correct estimation of presenteeism (recent attempts attempted to link it to health [71,72]), and the implementation of job-dependent multipliers. For now, it seems best to follow guidelines as much as possible, but to use sensitivity analyses to assess the effects of different valuation methods and corrections. For intangible effects, awareness (when relevant) is the starting point, but methodological work is needed to see how these can be consistently included in the decision-making process. Effects of the legal and fiscal context are in less need of methodological work, but the quality of economic evaluations and models would benefit from more thorough investigation and reporting of contextual factors by researchers performing economic evaluations.

The challenges of economic evaluation in OHS do not restrict themselves to the three elements discussed above. As indicated in the introduction, the measurement and attribution of effectiveness in OHS is particularly challenging, and often requires creative methodological solutions. Next, the loss of a colleague, friend, or family member can have substantial psychological consequences for the social environment of an employee, but it is unclear whether and how this should be taken into account. Normative standpoints also often remain implicit in analyses. For instance, the inclusion of productivity costs in economic evaluations can lead to a wider implementation of programmes that target productive individuals. By contrast, from an equity point of view, many populations that are less productive (e.g., retired employees, children) require more attention. However, it is as of yet not evident how to control for these effects and, more broadly, whether equity aspects should be considered altogether. Finally, barriers exist between researchers and business. For example, managers are not always well acquainted with economic evaluation methods (e.g., the difference between cost-effectiveness, cost–utility, or cost–benefit analyses), and have more affinity with simple summary measures (return-on-investment, payback period, benefit–cost ratio) that focus upon monetary consequences, while their use has substantial drawbacks [73,74,75]. Since these topics, as well as the three elements discussed above, build upon multiple domains—(health) economics, occupational health and safety, risk analysis, psychology—multidisciplinary research is key to work towards solutions.

## 5. Conclusions

In summary, general guidelines on economic evaluation remain largely applicable to the field of OHS, but researchers should pay attention to the specific challenges listed in this and other articles [8,15]. To ensure economic evaluations in OHS adequately inform decision-makers (especially employers), future methodological work should continue research on productivity, intangibles, and legal/fiscal effects, and work towards standardised methods of including them in economic evaluations, either directly or indirectly. We hope this article can help to generate more research in this direction.
